# Anger and the *Mahaabhaarata*

**DOI:** 10.4103/0019-5545.33265

**Published:** 2007

**Authors:** Ajit V. Bhide

**Affiliations:** Dept. of Psychiatry, St. Martha's Hospital, Bangalore - 560 001, India

The *Mahaabhaarata,*[1,2] the ancient Indian epic, has enlightening verses about a number of, perhaps all, issues that affect our daily lives. It is replete with examples of how damaging negative emotions can be.

Anger, particularly, is well exemplified and discussed in this epic.

“Real manliness,” it is stated in the first chapter, the *Aadi parva,* “lies in shedding anger much like the serpent casts off its worn skin against *kshamaa*”. There is a telling pun here, for “*kshamaa*” means forgiveness, as well as the Mother Earth. The serpent uses the friction generated in rubbing on the Earth to shed its skin; Forgiveness helps shed anger.

The example of the devastating effects of ire as portrayed in the *Mahaabhaarata* that stands out in my mind is Dhritaraashtra's wrath when his sons are defeated in the final war at the hands of the Pandavas. Soon after that victory, the Pandavas go to meet the old patriarch, their uncle. This uncle, Dhritaraashtra, calls upon the second Pandava, Bheem of legendary physical strength, who has killed his son Duryodhana, to come closer to be embraced, ostensibly to be congratulated on the victory. Krishna, ever the wily and wise, holds Bheem back. Instead, he places before the blind Dhritaraashtra, a solid metal statue of Bheem and the vigorous hug of the old man reduces the statue to rubble. Such is the vigor not only of his hug, but also of the anger that burns inside him. Later, when Yudhisthira bends to touch the feet of Dhritaraashtra's Queen, Gandhari, her partially covered vision falls but on his fingernails, which smoulder and turn black. The mother's anger also is very profound and literally burning.

Another insight into the cussed pervasiveness and perseverance of anger has been cited by Shamsundar (2006).[3] Much earlier in the epic when the princes-the Kauravas and the Pandavas are in their educative years under the tutorship of Dronaachaarya, the Guru gives the pupils assignments. On one such occasion, he enunciates ten principles that he wants the pupils to learn. The next day, when the Guru asks the students what they have learnt, all but Yudhishthira parrot out the enunciated principles. When the latter is asked, he states that he has learnt the first principle, but is not yet thorough with the second. Dronaacharya, less than exemplary himself in the control of anger, is enraged and begins to thrash the eldest Pandava. The prince takes the thrashing in a composed fashion. Seeing him so composed, Dronaachaarya suddenly realizes that something may be amiss. Pausing, he ponders, “This future King who could get me executed with just one order, is unperturbed by my wrath…. The first principle I taught him was to always be truthful; the second was to control one's temper…!” The teacher immediately realizes his folly and senses the Prince's sincerity in truly learning a principle.

The guru embraces the Prince and compliments him on his sincere learning of two principles, whereupon the Prince responds, “Sir! I haven't really mastered the second at all. But the first I am evincing even now. Just now as you were beating me, my ire was ignited and I have really struggled to keep it under control.”

Dronaacharya has a near-fatal flaw in his own temper. With two students he loses it; on each occasion because he feels cheated by a pretender pupil: Karna as well as Ekalavya.

Later in the final war, on opposing sides, when the ‘virtuous’ Yudhishthira tricks Dronaacharya into the erroneous belief that the latter's son Ashwatthaamaa is dead, the Guru resignedly gives up on life. His son, the self same Ashwatthaamaa, then is so enraged he practically wipes out all direct progeny of the Pandavas. This earns him a curse of an imperishable life of restless wandering.

In the third chapter of the Mahaabhaarata, the *Vana parva*, Yudhishthira is taunted by his Queen Draupadi. Draupadi's wrath at the lustful, lewd advances and innuendos of the Kauravas has become proverbial.

She urges him to take revenge on the evil Duryodhana, who has schemed against the righteous Pandavas and repeatedly personally humiliated her. In response, Yudhishthira delivers a treatise (in speech) to her on the value of forgiveness as opposed to the dangers that anger can engender.

Let us just look at the passage on anger (this is an adaptation by me of a well known translation by Ganguli):[4]

“Peculiar is anger for it both slays and protects; and peculiarly it begets all prosperity as well as adversity. For conquest of anger cannot but lead to prosperity and giving way to anger allows it to heap on you adversity. Destructive is anger to every living creature and so makes a sinner of man. The angry man hurts his elders and betters and fails to distinguish between what should be said and what should not…

“No act, no word, seems taboo for the angry one and in its grip will he slay indiscriminately. It is known that anger invites premature death. Knowing these dangers, the wise strive to gain control over anger. Forestalling wrath and not reacting to an angry man is like a physic against fear and one who manages such control, is like a physician to both, himself and the angry man.

“If a weak man persecuted by others, foolishly becomes angry towards men that are mightier than he, he then becomes himself the cause of his own destruction. And in respect of one who thus deliberately throws away his life, there are no regions hereafter to gain. Therefore, it has been said that a weak man should always suppress his wrath. And the wise man also who though persecuted, suffers not his wrath to be roused, enjoyeth in the other world, having passed his persecutor over in indifference. It is for this reason has it been said that a wise man, whether strong or weak, should ever forgive his persecutor even when the latter is in the straits. It is for this that the virtuous applaud them that have conquered their wrath. Indeed, it is the opinion of the virtuous that the honest and forgiving man is ever victorious….

“Truth is more beneficial than untruth; and gentleness is more beneficial than cruel behaviour. They that are regarded by the learned of foresight, as possessed of true force of character, are certainly those who are wrathful in outward show only. Men of learning and of true insight call him to be possessed of force of character who by his wisdom can suppress his risen wrath.

“Therefore, the man possessing force of character should ever banish wrath to a distance. The man that is overwhelmed with wrath acquires not with ease generosity, dignity, courage, skill and other attributes belonging to real force of character. The one who forsakes ire, becomes a true possessor of zest; one who is enslaved to anger possesses but a mockery of energy; that too which fails him in the hour of need.

“The turbulence of anger wrongly is considered energy by the ignorant….

“Wrath, however, has been given to man for the destruction of the world. The well behaved must learn to banish anger. Even one who has abandoned the excellent virtues of his own order, it is certain, indulges in wrath. If amongst men there were not persons equal unto the earth in forgiveness, there would be no peace among men but continued strife caused by wrath. If the injured return their injuries, if one chastised by his superior were to chastise his superior in return, the consequence would be the destruction of every creature and sin also would prevail in the world…

“If the man who has ill speeches from another returns those speeches afterwards; if the injured man returns his injuries; if the chastised person chastises in return; if fathers slay sons, sons slay fathers and if husbands slay wives and wives slay husbands; thus would there be no birth and no human race if anger prevails. If the king also gives way to wrath, his subjects soon meet with destruction. Wrath, therefore, has for its consequence the destruction and the distress of the people. And because it is seen that there are in the world men who are forgiving like the Earth, it is therefore that creatures derive their life and prosperity.”

This piece though insightful does have as much pragmatism as the extolling of virtue. One must notice that it is advised that the weak, particularly, need to guard against getting angry!

Two other references to anger in the many stories of this epic:

When the Princess Ambaa kidnapped by the regent, Bheeshma, for the Prince Vichitraveerya confides to him that she is in love with another, he sends her back with honour. Rejected by her lover upon her return, she turns to Bheeshma, asking that as he was responsible for her fate, he ought to marry her. Bheeshma's refusal to budge from his vow of celibacy enrages Ambaa to swear revenge; she immolates herself and is reborn as a male, who becomes the charioteer Shikhandi, instrumental in Bheeshma's death during the final battle.

When the Pandavas in the course of their wandering are faced with dire thirst, they one by one seek to take the water of a lake, which they discover is being guarded by a *Yaksha* (sprite). Each one of the younger four brothers refuses to follow the sprite's condition that the Pandava must answer his questions before partaking of the water and each in turn perishes. The sagacious Yudhishthira does answer the questions (which are an essay in wisdom by themselves) and is rewarded with water and by the revival of his deceased brothers. A vital insightful answer he provides is to the *Yaksha's* question, “Who is man's worst enemy?” Yudhishthira's prompt answer is “His own anger”.

The final word on anger of course is from Shri Krishna, in the second chapter, *Samkhya Yoga* of the *Bhagvad-Geeta* (itself part of Chapter 6, the *Bheeshma parva*):

***“Brooding on the objects of the senses, man develops attachment to them; attachment begets desire and from desire arises anger….***

***“Anger then begets delusion, delusion befuddles the memory and confused memory corrupts reason. Thence arises total ruination.”***

There are some other gems too on the theme of anger: “The man who is totally shorn of anger in the face of insults, is possibly impotent.”[5,6] (Chapter 2, *Sabhaa parva*)

“The learned and the ignorant alike, if caught in the web of lust and ire, are easy prey for the woman to set them on the wrong path.” (Chapter 13, *Anushaasan parva*)

In studying quotations from the *Mahaabhaarata*, it is important to never forget that it is written in story form and every noteworthy remark is not from the virtuous or the heroic; some witty and acerbic gems come from the evil ones too.

The Mahabhaarata has been transmitted down generations with many local embellishments and variations that have enriched its thought-provoking content.
